# Research on Determinants Affecting Users’ Impulsive Purchase Intention in Live Streaming from the Perspective of Perceived Live Streamers’ Ability

**DOI:** 10.3390/bs14030190

**Published:** 2024-02-28

**Authors:** Jun Chen, Junying Luo, Tian Zhou

**Affiliations:** School of Information Management, Wuhan University, Wuhan 430072, China; luojunying@whu.edu.cn (J.L.); zhoutian0113@163.com (T.Z.)

**Keywords:** live-streaming e-commerce, impulsive purchase, affective distance, affective trust, perceived live streamers’ ability

## Abstract

As an innovative marketing pattern, live-streaming e-commerce supplies advantages over traditional e-commerce in stimulating impulsive purchases. This study developed a theoretical model that examines how perceived live streamers’ abilities (perceived live interaction ability and perceived linguistic persuasion ability) affect impulsive purchase intention based on interaction theory, affective distance theory, trust theory, and Aristotle’s rhetorical appeals. We conducted empirical research through a survey questionnaire to verify the effectiveness of the model. A total of 330 valid samples were gathered from live-streaming users, and partial least squares–structural equation modeling (PLS-SEM) was employed for data analysis. The results indicate that perceived live interaction ability, encompassing responsiveness, entertainment, and personalization, significantly impacts affective distance. Among the four dimensions of perceived linguistic persuasion ability, emotional contagion significantly influences affective distance, whereas expertise, logic, and morality significantly affect cognitive trust. Both affective distance and cognitive trust promote consumers’ impulsive purchases through affective trust. Our research findings provide theoretical and practical recommendations for live-streaming platforms and merchants engaged in live marketing.

## 1. Introduction

The webcasting industry is progressively maturing with the continuous advances of information technology in the 21st century, which has enabled live-streaming e-commerce development. According to China’s published data, as of June 2023, the overall number of live-streaming consumers stood at 526 million, which constitutes 48.8% of Chinese web users, and the cumulative number of open anchor accounts exceeded 1.5 billion [1,2]. Live streaming facilitates online dealings between streamers and consumers, promoting live participation and enabling instant purchases. Impulse buying is a prevalent consumer behavior in live streaming. In accordance with the report conducted by the CCA, approximately 50% of Chinese consumers consider impulsive purchases during live streaming to be common, and almost 70% of respondents said they are likely to purchase products recommended by the live streamer, even if those products were not initially planned for purchase [3]. This means that live streamers can moderately influence users’ impulsive purchases of goods or services. The impulsive purchasing made by consumers is a complex process of psychological change, and is an emotional decision made by users after being stimulated by various factors in a short period. Therefore, grasping the psychological process and emotional basis beneath the formation of impulsive purchase intention, alongside a particular emphasis on the impact of the live streamer, assists in summarizing the characteristics of consumer impulsive purchasing behavior and fosters the sustainable advancement of live-streaming e-commerce.

The central theme of investigations on users in live streaming has consisted of examining their behavioral intentions and the components that govern their engagement, viewing, and buying behaviors. However, limited attention has been given to impulsive purchase intentions and the underlying determinants. Several researchers have undertaken research on the determinants of consumer usage and purchase, including the effects of marketing [4], product quality [5], consumer motivation [6], and other factors. Related studies have confirmed that the live streamer is the key to influencing users’ e-commerce purchase intentions and behaviors [4,5]. Nevertheless, the majority of research purely examines the impact of the streamer’s personal characteristics on user behavior, lacking in firmly investigating user perception. In live streaming, user perception is derived from the interactions between internal states (such as needs, motives, and emotions), environmental features, etc., which reflect the psychological state during live interactions [7,8,9]. Thus, live-streaming user perception research helps explore the internal psychological mechanisms underlying the formation of impulsive purchases.

As opposed to the traditional online marketing model, the ability of the live streamer is reflected in communicating with users through two-way communication in real time [10], stimulating user behavior by leveraging interactive and persuasive skills to evoke their emotional state. Consumer impulsive buying is believed to be closely related to emotional state [7]. During live-streamed sales, streamers mainly stimulate the user’s emotions through high-intensity and diverse interactions [11] and employ persuasive language expressions to effectively evoke emotional responses from users [12], thereby achieving the objective of promoting impulsive consumer purchases. Therefore, clarifying the mechanism of perceived live interaction ability and perceived linguistic persuasion ability on impulsive purchase intention is essential. Perceived live interaction ability depicts how consumers perceive streamers’ ability to lead real-time interaction, emphasizing the extent to which the streamer is instrumental to the user’s interaction experience. Previous studies have found that interaction positively promotes users’ attitude change and live-streaming engagement [11,13]. Meanwhile, the interaction between streamers and consumers affects purchase intention through mediators such as users’ emotional state and trust [14]. Thus, perceived live interaction ability helps to explain live-streaming users’ emotional changes and purchase intention [15,16]. Perceived linguistic persuasion ability reflects the users’ perception of streamers’ ability to persuade them to purchase products through verbal expressions. Prior research has applied the persuasion theories of Aristotle and Hovland, pointing out that linguistic persuasion can influence people’s decision-making through cognitive and affective reactions [12,17,18]. The persuasive ability and linguistic styles of the live streamer are critical factors in determining the sale results [12,19], and the relationship between perceived linguistic persuasion and impulsive purchasing needs to be further explored.

The mechanism of how perceived live interaction ability and perceived linguistic persuasion ability impact impulsive purchases during live streaming remains unclear. However, previous user behavior studies have shown that emotion drives impulse buying behavior [20]. Online impulse buying is determined by affective response, and contextual factors indirectly influence impulse buying through an affective reaction [9]. User-affective reactions in the shopping context are triggered by positive emotions such as enjoyment and pleasure [7]. During live shopping, live streamers engage in a diverse range of captivating and interactive experiences with users, fostering deep emotional connections. Previous research has demonstrated that social interaction positively influences users’ emotional perceptions [21], establishing a stronger emotional bond between streamers and consumers. Such closeness can stimulate impulsive purchases [22,23], which lessens the psychological distance between live streamers and consumers. Affective distance is an important dimension to measure the psychological distance at the emotional level, describing the degree of users’ perception and experience of streamers’ emotions. Incorporating the affective distance variable enables a deeper examination of the role of affective reaction between live interaction and impulsive purchasing from the perspective of consumers’ emotional changes.

Trust profoundly influences people’s purchase intention [14,24], which mainly includes two dimensions: cognitive trust and affective trust [25]. Specifically, cognitive trust has been described as a rational evaluation of whether individuals, groups, and organizations can be relied upon, considering factors such as professional competence, reliability, and similarity, whereas affective trust is an emotional connection that develops via mutual concern and interpersonal contact [26]. During live streaming, streamers use different linguistic persuasion modes, such as utilizing logical or rational elements in language expressions to convince consumers [17], exhibiting personal moral qualities such as integrity [27], as well as providing users with professional knowledge explanations, which influence users’ perceptions of the streamers’ professionalism, trustworthiness, and other abilities. Persuasive messages in live streaming can affect users’ attitudes and credibility evaluations to a certain extent, thus promoting users’ perceived trust in the streamers [28,29]. Moreover, the emotional elements of live streamers’ linguistic persuasion influence users’ emotions, providing them with a profound emotional experience and satisfying their emotional needs. It is easier for users to develop emotional intimacy, hence enhancing affective trust [30] if their needs for emotional experience are satisfied. Prior studies have established that cognitive trust is the underlying basis of affective trust, which indirectly affects impulsive purchase intention through affective trust [26]. Therefore, adapting affective and cognitive trust may deepen our understanding of the trust basis forming impulsive purchase intention, which emphasizes establishing an emotional tie between streamers and users.

In summary, this research aims to investigate the determinants affecting impulsive purchase intention by examining the live streamer’s abilities. We incorporate affective distance and trust theory to identify critical factors of user’s impulsive purchase intention. The remainder of the paper is structured as follows: Section 2 includes literature reviews on interaction, affective distance, trust, and impulsive purchase intention, explaining the pertinent and application value. In Section 3, we develop the theoretical model and establish the research hypotheses building upon related studies. Then, we illustrate the research methods in Section 4 and report data analysis and model validation results in Section 5. This study gathered samples using an online questionnaire and examined the hypotheses employing partial least squares–structural equation modeling (PLS-SEM). Finally, Section 6 discusses the research findings, delivers theoretical and practical implications, identifies the limitations, and proposes potential for further improvement. The results of our research will provide theoretical and practical proposals for live-streaming platforms and merchants.

## 2. Literature Review

### 2.1. Interaction

Prior studies of interaction focus on e-commerce, social networks, information systems, mobile platforms, etc. Burton and Soboleva defined interpersonal interactivity as “communication between people and organizations” [31,32]. In the field of live-streaming user behavior research, interaction theory has been widely used. Kai et al. implied that in live streaming, social interaction is an essential factor in establishing interpersonal relationships between streamers and users and promoting user behavior [13]. Zhang et al. suggested that interaction can enhance consumer trust and define interaction into subcategories such as active control and synchronicity [11]. Related studies demonstrated that interaction positively affects users’ emotional changes. As an illustration, Li et al. considered that the greater the interaction between viewers and streamers, the more likely viewers will be prone to constituency emotional attachments [21]. Kang et al. proposed that interactivity can positively impact mobile users’ emotional engagement, affecting user behaviors [15]. During live streaming, the connotation of interaction is prosperous. Live interaction is a representative way of information exchange, typically employing the “live and bullet comments” form of expression. Consequently, an immediate channel for disseminating information is established between streamers and viewers. Juxtaposed to traditional e-commerce shopping, the valuable upside to live-streaming e-commerce lies in the fact that streamers provide personalized and customized service instantly according to the user’s needs, such as specific answers and detailed product explanations. In addition, the entertainment experience in live streaming not only helps provide users with entertainment value, but also creates a pleasant shopping atmosphere, stimulates information exchange, and ultimately promotes impulse buying behavior.

### 2.2. Affective Distance

Psychological distance theory has been extensively applied in social communication and business marketing research [33,34,35]. Affective distance is derived from psychological distance, serving as a dimension of perceived emotion within the framework of psychological distance. Affective distance research in psychology focuses on emotional intensity and emotional arousal [36,37], which measures the extent to which subjects emotionally deviate from the target event or topic [38]. In communication studies, affective distance refers to the differences in emotional expression and perceived emotion between mass communicators and their audiences [38], and affective distance is influenced by the perception of power and interpersonal connection between the two parties [39]. Inadequate study concerns affective distance theory in live streaming. Only a handful of investigations demonstrated the applicability of psychological-distance-related theories in live e-commerce research [32,34,40]. Nevertheless, relevant studies have not explored, in depth, the antecedent variables that change the affective distance between the streamer and the user and the influence of affective distance on impulsive purchasing behavior. Psychological theory indicates that the closeness of the communicating parties at the emotional level depends on the experience and sharing of emotions [41,42], which means that the proximity of the streamer and the user at the emotional level depends on their emotional communication and experience. There is a wide range of emotional interactions between streamers and consumers in live streaming, which is mainly manifested in the live streamer’s ability to influence consumer perception through interaction and language so that they understand the streamer’s emotions, attitudes, goodwill, and integrity [24]. Affective distance can describe the degree of user perception and experience of the live streamer’s emotions. As the affective distance between the streamer and the user becomes close, the perception of the streamer’s emotion deepens, facilitating emotional resonance. Therefore, this study utilizes a perspective of affective distance to explore the deep emotional facets that affect impulsive purchase intention.

### 2.3. Trust Theory

Trust theory is commonly used to investigate the mechanisms influencing user behavior and online purchase intention [5,43]. Scholars have explored the antecedent variables of consumer trust and comprehensively summarized the relevant influencing factors from the perspectives of online merchant characteristics, environment, interaction, etc. [11,43,44]. McAllister proposed that trust could be defined as two distinct forms: cognitive-based trust and affective-based trust [45]. Based on prior studies, cognitive trust is the belief that one can rely on others’ expertise and abilities [46,47]. Cognitive trust includes the confidence an individual has in the other individual’s attributes in a certain scenario, based on beliefs concerning the other’s reliability and dependability [45]. In contrast, affective trust measures the level of attention and attachment of the trusting party to the trusted party [45], reflecting the emotional bond and connection between the parties. Related research has considered that the emotional bond between users and service providers could possess a favorable impact on their motives and actions, such as consumer loyalty, behavioral commitment, and behavioral intents [21].

Prior scholars have identified the significance of cognitive trust and affective trust in users’ e-commerce purchases [46]. Zhang et al. concluded that the cognitive trust of consumers in online retailers influences affective trust, which subsequently promotes purchase intention [48]. Chen et al. concluded that impulsive purchasing is determined by affective trust in the recommender and emotion towards the recommended product, with affective trust being one of the direct determinants of impulsive purchase intention [26]. Lu et al. observed that online streamers’ performance could stimulate consumers’ emotions and foster affective trust, thereby boosting consumers’ purchase intention for recommended products [49]. Investigations have summarized the factors that determine cognitive trust and affective trust in connection with products and services, e-commerce streamers, platforms, consumer characteristics, etc. Service quality, streamers’ competence and expertise, credibility, and reputation are key factors that influence consumers’ cognitive trust, and these variables impact user evaluation, ultimately affecting purchase intention [26,30,46]. For affective trust, studies have been undertaken to determine the antecedent factors that affect emotional connection, including intimacy, online performance, and interaction [30,46,49]. Previous studies have drawn attention to the mediation effect of affective trust in the relationship between cognitive trust and users’ purchase intention [48]. This research concentrates on cognitive trust and affective trust, examining how trust promotes the impulsive purchase intention of live-streaming users. Furthermore, we consider perceived streamers’ ability a crucial factor influencing trust.

### 2.4. Aristotle’s Rhetorical Appeals

Persuasion is the process of altering other’s attitudes and actions in line with the persuader’s intended effect. Studies on Aristotle’s rhetorical appeals pointed out that the process of persuasion essentially involves applying the three modes of persuasion, which include Ethos, Logos, and Pathos [50]. Among them, Ethos (appeal to credibility) means that the persuader obtains the audience’s trust in personality traits by showing knowledge and morality. Logos (appeal to logic) implies that the persuader emphasizes logic in the process of persuasion and proves their point of view using logical arguments. Pathos (appeal to emotions) involves the persuader arousing the audience’s emotions, causing them to empathize, and influencing rational judgments. Using different linguistic persuasion modes to describe the same content can bring different persuasive results. Previous studies of Aristotle’s rhetorical appeals concentrated mainly on the fields of politics [17], tourism economics [18], and e-commerce platforms [12].

In live streaming, streamers use different linguistic persuasion modes to convey product information to the users and influence their attitudes, thoughts, and behaviors, which ultimately persuades them to purchase. This approach matches effectively with the definition of persuasion. Streamers use different linguistic persuasion modes, such as guaranteeing the quality of the goods with credibility, elaborating detailed information, and introducing the discount information in a clear and lively tone, which correspond to the rhetorical triad of the three modes of persuasion. Therefore, it is rational and imperative to apply Aristotle’s rhetorical appeals as a basis for defining the persuasive ability of live streamers. We consider that live streamers’ persuasive ability refers to persuading consumers to buy products through different linguistic modes. According to Aristotle’s rhetorical appeals, the perceived linguistic persuasion ability includes four dimensions, choosing morality and expertise to describe the two aspects of appeal to Ethos, respectively, using emotional contagion to represent the appeal to Pathos, and employing logic to denote the appeal to Logos. Table 1 presents the constructs and definitions.

### 2.5. Impulsive Purchase Intention

Impulsive purchasing is an unplanned purchase decision triggered by various stimuli [53]. Consumers confronted with external stimuli while shopping exhibit intense affective responses, which lead them to make impulsive, irrational, voluntary, and instant purchases [54]. Kimiagari and Malafe proposed that two kinds of external and internal stimuli affect consumers in the decision-making process of online impulse buying; specifically, external stimuli (containing product features, store features, marketing stimuli, etc.) are objective factors that users do not control, while internal stimuli are within personal control, including the unique characteristics of the users [55]. Compared to traditional shopping, live streaming offers a higher probability of impulse buying to a certain extent. It further integrates e-commerce, social activities, and situational factors, influencing users’ cognition and emotional perception [56]. Currently, research on live-streaming impulsive purchases is still developing and has yet to be fully explored. Only some studies examined the factors influencing consumers’ impulsive purchases from both live-streaming consumer and supplier perspectives [43]. Previous scholars have distinguished the relationship between impulsive purchase intention (the urge to buy impulsively) and actual impulse buying behavior, arguing that impulsive purchase intention is a sudden, powerful, and persistent urge to buy an item immediately, while impulse buying behavior is the subsequent action undertaken [57,58]. Since measuring actual impulse buying behavior is challenging [7], most studies have used impulsive purchase intention (the urge to buy impulsively) as a proxy for actual buying behavior [9,26,59]. Thus, we choose impulsive purchase intention as the observed variable of consumer behavior. This research aims to investigate features associated with impulsive purchasing in live streaming, analyze the influencing mechanism of users’ impulse purchase intention from multiple perspectives, and excavate important contributing factors.

## 3. Research Model and Hypotheses

### 3.1. Construction of Theoretical Framework

Combining interaction theory and Aristotle’s rhetorical appeals, this study distinguishes the perceived live streamers’ ability as perceived live interaction ability (responsiveness, entertainment, personalization) and perceived linguistic persuasion ability (emotional contagion, expertise, logic, and morality).

From the perspective of perceived live interaction ability, previous studies have revealed that interactive experiences trigger users’ emotional responses and impulsive behaviors [15,16,60]. The increased interaction between e-commerce streamers and users can promote users’ positive attitudes and perceived value toward live streaming [61,62]. The immediate interaction between streamers and users can boost the flow of information and emotions in live streaming, effectively reduce perceived risks, and encourage the occurrence of user behaviors [60]. Therefore, perceived live interaction ability is applicable to measure the extent to which the interactive ability of streamers has a role in user perception. In addition, many studies have summarized the characteristics of interaction from the basic dimensions of synchronization, responsiveness, personalization, etc. Entertainment is also a prominent feature of live streaming, positively impacting users’ attitudes [61]. Therefore, we chose perceived live interaction ability as the first dimension of perceived live streamers’ ability and concluded perceived live interaction ability from three perspectives: responsiveness, entertainment, and personalization. Responsiveness reflects the user’s perception of the streamer’s ability to respond quickly and effectively to user demands, including offering immediate feedback and actively responding to inquiries [32]; personalization measures the user’s perception of the streamer’s ability to satisfy users’ needs and to deliver tailored services [13,32]; and entertainment reflects the user’s perception of the streamer’s ability to pleasure users with enjoyable activities [32,61].

From the perspective of perceived linguistic persuasion ability, the impact of e-commerce streamers’ persuasive ability on impulsive purchasing behavior manifests in users’ cognitive and emotional states [12,17,18]. In terms of user cognition, when the live streamer shows expertise, logic, and morality, these cheerful and persuasive abilities help to build trust, thus affecting user behavior [18]. At the emotional level of users, the live streamer realizes emotional transmission to users through emotional infection and uses persuasive, vivid language to obtain users’ identification and emotional resonance, thus achieving an apparent effect [17]. Therefore, we choose perceived linguistic persuasion ability as the second dimension of the perceived live streamers’ ability. As mentioned, live streamers’ persuasive ability refers to persuading consumers to buy products through different linguistic modes. Perceived linguistic persuasion ability is essential to users’ cognitive and psychological states. Combined with the research on Aristotle’s rhetorical appeals, this study summarized the perceived linguistic persuasion ability from the four dimensions of emotional contagion, expertise, logic, and morality. Emotional contagion reflects live streamers’ ability to convey emotions through language and cause emotional resonance in consumers [51,52]. Expertise refers to live streamers’ knowledge level and ability correlated to commodity trading conveyed by linguistic expression [46]. Logic refers to the streamers’ ability to supply a complete presentation structure and clear demonstration process through logical argument [17,28]. Morality refers to the ability of live streamers to illustrate their moral quality and shape honest images through language expression [27].

This study combines the affective distance theory and the trust theory to analyze the impact of perceived streamers’ abilities further. In prior research on live-streaming user behavior, researchers have mainly used user motivation, perceived value, and perceived usefulness as mediating variables between various stimuli and impulsive purchasing behavior [4] and rarely examined the role of affective variables. Affective factors are considered to be the key to the research on consumers’ online impulse buying behavior [20,54]. Affective distance describes how users perceive and experience the emotions of e-commerce streamers and plays a vital part in the analysis of customers’ internal emotional states and psychological dynamics. Therefore, it is appropriate to explore the streamers’ abilities on impulse purchasing from the perspective of users’ emotional perception. Moreover, related scholars have demonstrated the significance of trust in live-streaming purchasing, encompassing users’ cognitive and affective dimensions [11,63]. However, the exploration of trust in live streaming needs to be completed, with a limited investigation into the connotation, antecedent variables, and mechanisms. Furthermore, current research still requires an examination of the relevance of the connection between consumers’ cognitive trust and affective trust toward live streamers. In this study, the framework of user trust in live-streaming e-commerce was established based on the literature associated with cognitive trust and affective trust. Cognitive trust develops via assessing the qualities of others [26,46], which mainly includes the dimensions of competence, benevolence, and integrity [48]. Affective trust is a form of reliance on others that is emotionally driven, characterized by perceived relationship strength and emotional intimacy [30,46].

In summary, considering perceived live streamers’ ability (including perceived live interaction ability and perceived linguistic persuasion ability), we developed a model of influencing factors of impulsive purchase intention that integrates affective distance theory and trust theory. Further, the research hypotheses are discussed in the subsequent sections.

### 3.2. Research Hypotheses

#### 3.2.1. Trust and Impulsive Purchase Intention

(1) Affective trust and impulsive purchase intention

Affective trust refers to an emotional bond or connection between viewers and live streamers based on how much the streamer cares about the viewers. In pertinent research, trust is often regarded as an essential user belief, including cognitive and affective trust in merchants and products, which positively affects user purchasing behavior [64]. Live streaming enriches users’ shopping experience, fostering perceived enjoyment and pleasure so that the positive emotional state can be the basis for forming users’ impulsive purchase intention [7,26,55]. Affective trust reflects a positive emotional connection, and affective trust in e-commerce streamers can stimulate consumers’ impulsive purchase intentions [26]. In live streaming, when there exists emotional dependence and a connection between users and streamers, users tend to be more willing to buy the products streamers recommend, even if the item is not in the range of planned purchases [49]. Thus, we propose the following hypothesis:

**H1.**  *Users’ affective trust in live streamers is positively related to impulsive purchase intention.*

(2) Cognitive trust and affective trust

Cognitive trust is a rational assessment of whether a live streamer is trustworthy or not based on competence, professionalism, reliability, and other information in a live e-commerce situation. It measures the extent to which consumers believe or are willing to rely on a service provider’s competence and skills [25]. Multiple studies have demonstrated that the development of cognitive trust contributes to the establishment of affective trust. Zhang et al. noticed that cognitive trust, which is based on competence, benevolence, and integrity, can positively influence affective trust in online retailers [48]. Johnson and Grayson verified that cognitive trust serves as a foundation for fostering affective trust during the establishment of interpersonal trust [46]. Srivastava et al. demonstrated that cognitive trust is the foundation for affective trust, as users typically rely on their cognition and past experiences before forming affective trust [30]. Furthermore, a high degree of cognitive trust leads to a reduction in uncertainty, which, in turn, promotes users’ emotional attachment to the live streamers, resulting in affective trust [26]. Perception of the streamer’s personal traits (professionalism, morality, sincerity, skills, etc.) may help to enhance cognitive trust, as well as to form a favorable impression and recognition of the streamers, and this positive emotion will strengthen affective trust in the streamer [25]. Therefore, from combining the above discussion, we suggest the following hypothesis:

**H2.**  *Users’ cognitive trust in live streamers is positively related to affective trust.*

#### 3.2.2. Affective Distance

(1) Affective distance and Affective trust

Affective distance describes the degree to which users perceive streamers’ emotions. Previous studies indicated that alterations in psychological distance in online buying situations can influence consumers’ credibility assessments and trust relationships with service providers [34,40]. From the perspective of interpersonal psychology, reducing psychological distance decreases consumers’ self-defense mechanisms and fosters the user perception of genuineness, honesty, openness, and trustworthiness [32,33]. Affective distance variation in live streaming impacts the tightness of the emotional bond between the user and the streamer. Higher emotional intimacy between the two parties increases the potential for affective trust while facilitating the establishment of a solid emotional connection, as customers are more inclined to trust streamers who show empathy and concern [30]. Positive emotional feelings are the basis for establishing an emotional bond [65]. Live streamers who intuitively sell products can enhance consumers’ sentiments and feelings, stimulating users’ emotional resonance and fostering familiarity and intimacy with the streamers [21,32,41]. This may assist in establishing a reliable emotional connection [24] and encourage the development of affective trust. Thus, this study suggests the following hypothesis:

**H3.**  *Affective distance is positively associated with users’ affective trust in live streamers.*

(2) Affective distance and impulsive purchase intention

Relevant studies have revealed that users’ perceived psychological distance and social presence to streamers influence their emotional state and motivation [32,43]. Positive emotional factors are crucial for users to make impulsive purchases [54]. Enjoyment, impulsivity, pleasure, and arousal are the basic emotional reactions associated with online impulse purchasing [7]. When streamers describe and present products through friendly, enthusiastic, or humorous language and demeanor during live streaming, it helps to elicit consumers’ pleasure and arousal emotions, which positively affects purchase intention [49]. During a live purchasing scenario, when the user is near the streamer at an affective distance level, they may experience an aroused emotional state and have better access to emotional experiences from the streamer. Users are more receptive to impulsive purchases when emotionally close to the live streamer as they obtain sufficient emotional stimulation and are in a pleasant emotional state. The rich, dynamic properties of streamer behavior can carry powerful emotional stimulation, leading to affective responses [38], which may result in impulse purchases. Hence, we put forth the following hypothesis:

**H4.**  *Affective distance is positively associated with impulsive purchase intention.*

#### 3.2.3. Affective Trust as Mediator

Trust is a direct determinant that affects user behavioral intention with a significant favorable influence on promoting purchase intentions [5,24]. Meanwhile, psychological distance is considered to be crucial in forming consumer trust, and the reduction in user-perceived psychological distance positively enhances consumer trust [34,40]. Affective trust is one of the essential dimensions of trust, which describes consumers’ emotional dependence on live streamers formed by caring and interpersonal interactions [26]. Streamers affect consumers’ emotional experiences through interaction and persuasion to bring closer affective distance between them. Continuous emotional experience and perception promote the formation of consumers’ emotional dependence [21], which, in turn, affects consumers’ impulsive purchasing. Additionally, some scholars believe that among the different dimensions of trust, cognitive trust influences user intention through affective trust [26,30,46]. Parboteeah et al. posited that affective reactions have a more direct effect on users’ impulse buying urges than cognitive reactions [9]. Therefore, affective factors mainly drive the formation of impulsive purchase intention among live-streaming consumers [54] and, to a certain extent, lie more on affective trust [26]. In summary, this study suggests the following hypotheses:

**H5.**  *Affective trust mediates the relationship between affective distance and impulsive purchase intention.*

**H6.**  *Affective trust mediates the relationship between cognitive trust and impulsive purchase intention.*

#### 3.2.4. Perceived Live Interaction Ability

The interaction between streamers and consumers has been widely regarded as two-way, autonomous, and synchronous, and high-intensity interaction emerges as the most prominent distinction between live streaming and traditional e-commerce [11]. Besides this, live interaction’s uniqueness lies in its richness, timeliness, and entertainment value [13]. The increase in interaction promotes the viewer’s positive attitude, while the entertainment features of live streaming additionally influence the user’s attitude and perception [61]. We identified three dimensions of perceived live interaction ability: responsiveness, personalization, and entertainment, and then further analyzed its mechanism in users’ impulsive purchases.

(1) Responsiveness

Responsiveness describes the degree to which users perceive live streamers’ fast and effective responses. Responsiveness reflects the practical process of interpersonal communication between streamers and users, with both parties acting as the main participants in the interaction. Prior studies have revealed that active interaction makes consumers feel a higher level of social presence, increasing information and emotional exchange [14,24,43], thus enhancing users’ emotional experience of streamers. In the process of live interaction, streamers can not only intuitively and fully explain and display products, but also provide quick feedback to users’ questions and output effective results in real time [32]. Instant feedback interaction assists in creating a better emotional experience, which promotes users to be more attentive to the communication with the live streamer. The positive interaction of the two on the emotional level may further narrow the affective distance. Thus, we put forth the following hypothesis:

**H7.**  *Responsiveness is positively associated with the affective distance between live streamers and users.*

(2) Entertainment

Entertainment refers to the inherent pleasure consumers perceive when participating in the activities initiated by live streamers. Bosshart and Macconi defined entertainment experience as pleasure, stimulation, relaxation, and diversion [66]. In essence, live streaming is a kind of audio and video media that is entertaining. Previous studies have indicated that audiences hope to use media for entertainment and relieve pressure [61]. Real-time interaction fulfills users’ entertainment needs so that they can be pleased, engaged, and in a positive emotional state, after which purchase behavior can be implemented. Entertainment can also hold users’ attention and interest and significantly impact users’ emotional perception and satisfaction [61,67]. Through engaging interactions, live streamers transmit positive emotions to users and enrich users’ emotional experiences, thus narrowing the emotional distance between them more easily under the positive emotional state. Hence, we put forth the following hypothesis:

**H8.**  *Entertainment is positively related to the affective distance between live streamers and users.*

(3) Personalization

Personalization refers to live streamers providing personalized services to consumers to satisfy their specific requirements and maximize business opportunities. Personalized marketing strategies create a perception of being taken seriously, which establishes a sense of belonging to live streaming and eliminates the emotional alienation between users and live streamers [11,16,33], thus narrowing the perceptual and psychological distance between streamers and users at the emotional level. E-commerce streamers timely adjust live strategies according to the characteristics of different types of consumers and provide targeted and unique product solutions [11], which can obtain users’ favor and enhance closeness [32]. Streamers employ emotional expressions such as strong recommendations to comply with customized demands when providing personalized services to pass on positive emotions to users, which may shorten the emotional distance. Therefore, we propose the following hypothesis:

**H9.**  *Personalization is positively associated with the affective distance between live streamers and users.*

#### 3.2.5. Perceived Linguistic Persuasion Ability

(1) Emotional contagion

Emotional contagion reflects live streamers’ ability to convey emotions through language and cause emotional resonance in consumers. In live-streaming e-commerce, streamers frequently use emotionally rich, vividly expressed language to achieve the effect of emotional infection, accurately conveying their own emotions or motives to the user’s claims [17]. In the highly emotional atmosphere of live streaming, consumers are efficiently infected by the emotions of streamers, develop empathy towards them, and experience positive emotions akin to those expressed by streamers [68]. Live streamers utilize verbal descriptions and other methods to interact with users, arouse their emotions, and stimulate pleasant emotional experiences, thus achieving the effect of emotional infection [49]. Users are the receivers of emotions and, at the same time, have the ability not only to perceive the streamer’s emotions, but also to generate further positive emotional feedback due to the streamer’s linguistic description. Additionally, the linguistic persuasion style of streamers with strong emotional characteristics may be more easily perceived by users, leading to positive emotional states, which enables the shrinking of the emotional distance between these two parties. Thus, we put forth the following hypothesis:

**H10.**  *Emotional contagion is positively related to the affective distance between live streamers and users.*

(2) Expertise

Expertise refers to live streamers’ knowledge level and ability correlated to commodity trading conveyed by linguistic expression. According to Kelman, expertise depicts the degree to which the information receiver can perceive that the information or experience the information spreader provides is correct [69]. Live streamers’ expertise improves the quality of information and service, thus positively affecting user perceptions and attitudes [70,71,72]. During the e-commerce sales process, streamers’ skill in product explanation and concern for their audience assists users in perceiving rich professional information, saving time and energy, building customer trust in streamers [46], and ultimately making voluntary purchases. Therefore, we suggest a further hypothesis:

**H11.**  *Expertise is positively associated with users’ cognitive trust.*

(3) Logic

Logic refers to the streamers’ ability to supply a complete presentation structure and clear demonstration process through logical argument. In live streaming, logic describes whether the language expression of streamers is organized, clear-cut, and based on theories and facts [28]. When explaining products, experiencing demonstrations, and launching activities, the logical language of live streamers enables users to comprehend product information in detail. Furthermore, logical and concise language expression fulfills the needs and preferences of consumers for information acquisition while reducing perceived uncertainty [73]. When the streamer effectively communicates and presents their perspectives, users can not just comprehend the information conveyed by the streamer, but also spontaneously evaluate the expression of language, which impacts their satisfaction and cognition. Users’ faith in streamers might be enhanced when they recognize the utility of the streamers’ logical language. Thus, we put forth the following hypothesis:

**H12.**  *Logic is positively associated with users’ cognitive trust.*

(4) Morality

Morality refers to the ability of live streamers to illustrate their moral quality and shape honest images through language expression. When consumers believe that the streamers’ behavior is in line with moral standards and norms, they assume that the streamer will serve in an ethical way, thus affecting consumers’ attitudes toward the streamers [27]. In live streaming, the morality of streamers (such as integrity, friendliness, and sincerity) [74] delivered by real-time interaction and product display makes users realize that the streamers are trustworthy, thus reducing perceived risks, increasing psychological safety, and promoting users’ trust. In addition, users’ judgments of streamers’ moral qualities are mainly based on their moral standards, and the similar moral qualities between these two parties contribute to establishing the psychological connection of identity, thus enhancing cognitive trust [5,27]. This study suggests the following hypothesis:

**H13.**  *Morality is positively related to users’ cognitive trust.*

### 3.3. Research Model

**Figure 1:**
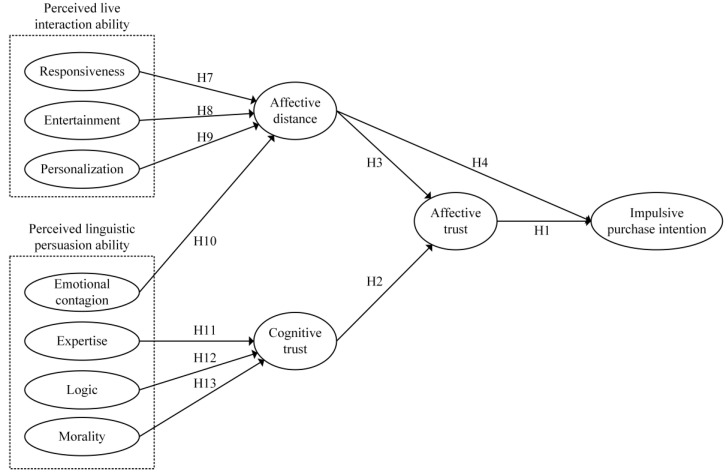
Research model.

As depicted in Figure 1, according to the previous theories and assumptions, this research developed a model of impulsive purchase intention.

## 4. Research Methods

### 4.1. Measures

The survey utilized a questionnaire of two distinct sections to gather empirical data. The first section was used to assess the demographic characteristics, which included age and gender. The second section contained all of the constructs. To ensure the questionnaire’s validity and maintain consistency between the Chinese and English scales, this study employed the strategy of translating the English questionnaire into Chinese. After the initial preparation of the questionnaire, participants who routinely participated in live-streaming e-commerce were contacted to partake in interviews. The research questionnaire was adjusted for this study, taking into consideration the results of the interviews, in terms of its phrases, content, and structure. The questionnaire was prepared alongside a Likert scale of five, with numerical scores of 1 to 5 equating to the degrees of agreement that span complete disagreement to complete agreement, respectively. With regard to the details provided in Table 2, the variable definitions and measurements utilized were derived from prior research, which was further adapted to the specific research background.

(1) Perceived live streamers’ ability

For perceived live interaction ability, we employed the mature scales developed by Xue et al. [32] to measure responsiveness, entertainment, and personalization. For perceived linguistic persuasion ability, the scale of emotional contagion was adapted from the studies of Reniers et al. and Shen [51,52]; the scale of expertise was aligned with Johnson and Grayson’s research [46]; the logic scale was adjusted with Chang [28]; and the scale of morality was derived from Zhou [27].

(2) Affective distance

This study measures affective distance based on Lu et al.’s scale [24]. Our study stresses the effects of live interaction and emotional perception on affective distance. The mature scale of psychological distance designs items from the perspectives of similarity, environmental atmosphere, social presence, etc. [34,75]. We consider affective distance as one of the important dimensions of psychological distance. Live interaction and emotional perception are crucial to narrowing emotional distance and enabling consumers to feel a high social presence, thus promoting emotional interaction [24,43]. Therefore, the scale was adapted for emotional experience and user perception to highlight the streamer’s influence. Meanwhile, we modified the research background of the established scale to conform better to the live-streaming context.

(3) Cognitive trust and affective trust

The cognitive trust scale was developed from McAllister’s mature scale [45]. Prior frameworks of cognitive trust generally contain aspects of competence, professionalism, sincerity, reliability, benevolence, etc. [30,46,48,76]. This study followed the previous maturity scale and measured cognitive trust regarding users’ assessment of the streamer’s professionalism, attitude, and trustworthiness. Among them, professionalism represents the streamer’s ability to complete the live streaming and facilitate user purchases. Attitude includes the streamer’s dedication and performance. Trustworthiness describes the user’s trust in the streamer’s professional ability, experience, and performance.

The affective trust scale was adapted from the well-established scale of Srivastava et al. [30]. In measuring affective trust, the streamer’s caring concern, serious-minded emotions, and positive responses to the user were mainly assessed, which is usually considered an essential emotional basis for forming affective trust.

(4) Impulsive purchase intention

Finally, the impulsive purchase intention scale was derived from Parboteeah et al.’s well-established scale on the urge to buy impulsively [9].

### 4.2. Data Collection and Samples

The questionnaire was pre-tested before formal data collection, and a total of 117 valid samples were gathered. We performed Exploratory Factor Analysis and Confirmatory Factor Analysis. Based on the results of these analyses, we found that some constructs were identified as problematic and were either adjusted or removed. The completed questionnaire contained 11 constructs totaling 39 items, each containing 3 to 5 items. The research data were collected in April 2022. Relying on the professional questionnaire survey platform Sojump and covering users of Taobao, Douyin, Kuaishou, etc., the research data were randomly gathered online. Hyperlinks of the questionnaire were positioned online, and only those users with live-streaming shopping experiences were targeted for data collection. Screening questions were included in the questionnaire to ensure that all respondents utilized various platforms. The participants received instructions to recall their live-streaming purchase experiences and complete the survey. After excluding the questionnaires that did not fit the study’s standards, a total of 330 valid survey responses were extracted.

The minimum sample size was checked to ensure that the sample had sufficient statistical capability. According to a rule of thumb, the minimum sample size for a PLS model should be equal to the larger of the following: (1) ten times the maximum number of formative indicators utilized to measure one construct; or (2) ten times the maximum number of inner model paths directed at a particular construct [77,78]. We collected a sample size of 330, which surpasses the minimum requirement and is considered sufficient as it exceeds ten times the number of constructs and inner model paths. Additionally, we completed data analysis of the samples employing partial least squares–structural equation modeling (PLS-SEM), which does not require the samples to be normally distributed and is suitable for small sample sizes [79,80]. Despite not restricting the normal distribution of the sample in PLS-SEM, we conducted the Shapiro–Wilk and Kolmogorov–Smirnov tests, finding that a portion of the data did not follow a normal distribution. Considering these concerns, we maintain that PLS-SEM is suitable for assessing the data in our examination.

Table 3 summarizes a variety of characteristics associated with the sample. There were 330 respondents, of which 147 (44.55%) were male and 183 (55.45%) were female. The research revealed a relatively equal distribution of male and female respondents. More than half of the participants were in the age spectrum ranging from 26 to 35 (*n* = 182, 55.15%). Furthermore, a considerable proportion held a bachelor’s degree (*n* = 301, 91.21%). Based on multiple industry reports on live-streaming e-commerce, Chinese e-commerce consumers born in the 1980s and 1990s comprise over 80% [81]. Regarding gender distribution, females account for around 54% of the total consumers, slightly more than males [82]. Our sample aligns with the given characteristics, ensuring the survey data are statistically reliable.

## 5. Results Analysis and Hypothesis Testing

### 5.1. Assessment of Measurement Model

To evaluate the common method biases, this research employed the IBM SPSS Statistics 22 program to execute Harman’s single-factor test to ensure the validity of the analysis results. Unrotated exploratory factor analysis indicated that the first factor extracted with an eigenvalue surpassing one explained 33.548% of the overall variance, less than the threshold of 40%, which passes the conditions.

Reliability is defined as the degree to which outcomes remain consistent when the measurement steps are repeated on the same item with the same measurement method. A reliability test can be employed to evaluate the dependability of the sample. In most studies, Cronbach’s alpha reflects the reliability well. We measured the reliability by estimating Cronbach’s alpha, which provided a sign above 0.7, meaning reasonable reliability, as shown in Table 4.

Validity tests are used to examine whether questionnaire items can effectively express the conceptual information contained in the variables, mainly including convergent and discriminant validity. The average variance extracted (AVE) ranges from 0.55 to 0.766, and the composite reliability (CR) spans from 0.847 to 0.908, as shown in Table 4, proving significant convergent validity. Furthermore, we evaluated discriminant validity by employing the Fornell–Larcker criterion method and cross-loadings. As illustrated by the results provided in Table 5 and Table 6, the items held sufficient discriminant validity.

### 5.2. Hypothesis Testing

**Figure 2:**
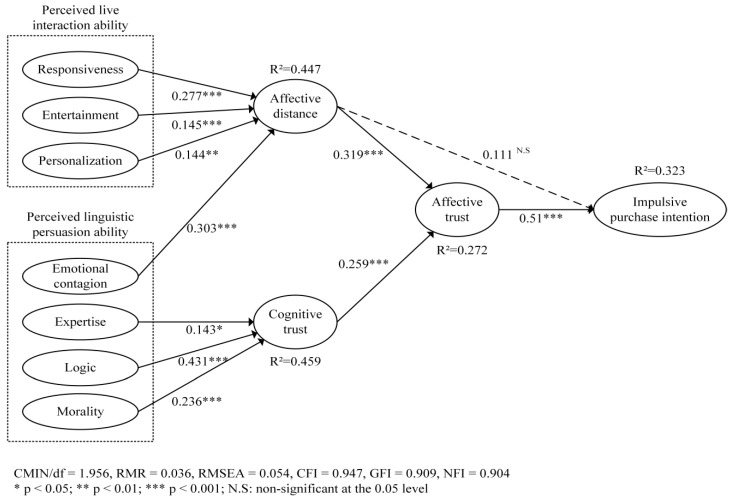
Research model running results.

Partial least squares–structural equation modeling (PLS-SEM) is suitable for validation and predictive capacity assessment [83,84]. The subsequent data analysis in this study was conducted through Smart PLS 3.33, a specialized statistical analysis software program. In our research, the structural equation model is constructed for hypothesis testing, which explains 32.3% of the variance of users’ impulsive purchase intention, as presented in Figure 2. The R^2^ of cognitive trust, affective trust, and affective distance are 0.459, 0.272, and 0.447, respectively, which met the criterion that the R^2^ is more significant than 0.2. CMIN/df (1.956), RMR (0.036), RMSEA (0.054), and CFI (0.947), as well as other indicators, satisfy the standards established. Thus, the model fitting results are acceptable.

The model analysis results indicate that affective trust (β = 0.51, *p* < 0.001) positively correlates with impulsive purchase intention, supporting H1. Cognitive trust (β = 0.259, *p* < 0.001) significantly and positively influences affective trust, and hypothesis H2 is supported. Affective distance (β = 0.319, *p* < 0.001) significantly affects affective trust, but has no significant effect on impulsive purchase intention (β = 0.111, *p* > 0.05). Thus, H3 is supported, while H4 is not supported. In terms of perceived live interaction ability, responsiveness (β = 0.277, *p* < 0.001), entertainment (β = 0.145, *p* < 0.001), and personalization (β = 0.144, *p* < 0.01) are significantly related to affective distance, respectively, with responsiveness having the most significant effect on affective distance, indicating that H7–H9 are supported. Among the four dimensions of perceived linguistic persuasion ability, emotional contagion (β = 0.303, *p* < 0.001) is positively correlated with affective distance, and expertise (β = 0.143, *p* < 0.05), logic (β = 0.431, *p* < 0.001), and morality (β = 0.236, *p* < 0.001) significantly influence cognitive trust, supporting H10–H13.

To further investigate the mediating effect of affective trust between affective distance, cognitive trust, and impulsive purchase intention, we applied the three-step method proposed by Baron and Kenny [76]. As provided in Table 7, the results indicate that affective trust completely mediates affective distance and impulsive purchase intention, and plays a partial mediating role between cognitive trust and impulsive purchase intention. Thus, H5 and H6 are supported.

## 6. Discussion and Implications

### 6.1. Discussion

Based on interaction theory, affective distance theory, trust theory, and Aristotle’s rhetorical appeals, we investigated the determinants of impulsive purchase intention and constructed the path of impulsive purchase intention formed by “perceived live streamers’ ability—affective distance—trust—impulsive purchase intention” and drew the following conclusions.

First, the results of this study show that perceived live interaction ability has a significant positive influence on bringing closer affective distance between live streamers and users. Affective distance is significantly positively correlated with affective trust, thus affecting impulsive purchase intention. This conclusion indicates that perceived live interaction ability positively impacts users’ emotional reactions in live-streaming e-commerce, which further involves impulsive purchase intention. That is, when streamers exhibit interaction ability with responsiveness, entertainment, and personalization in live streaming, it will contribute to narrowing the affective distance with consumers, facilitating the formation of affective trust, and ultimately influencing impulsive purchase intention.

Second, perceived linguistic persuasion ability (expertise, logic, morality, and emotional contagion) influences both affective distance and cognitive trust, among which emotional contagion promotes affective distance by awakening consumers’ positive emotions, making them feel pleasure, thus decreasing affective distance and generating affective trust in the streamers. In addition, expertise, logic, and morality are significantly positively associated with cognitive trust. This is mainly because in e-commerce live streaming, expertise, logic, and morality have an impact on consumer cognition. The professional and rich language expression, clear organization, and heightened moral level of the streamers make consumers aware of their credibility and enhance user cognitive trust towards them, which, in turn, enables consumers to develop affective trust in streamers, ultimately affecting their impulsive purchase intention.

Moreover, according to the mediating effect testing result, affective trust fully mediates the relationship between affective distance and impulsive purchase intention and partially mediates the relationship between cognitive trust and impulsive purchase intention. The result above fits with the expected outcomes of our investigation. We prove the impact between cognitive and affective trust, illustrating the critical role of trust variables on impulsive purchasing.

To sum up, our results reveal the intrinsic mechanism of perceived live streamers’ ability to impulse purchase intention, emphasizing the essential role of affective distance and trust. Earlier research suggested that live interaction between streamers and users decreases psychological distance [32]. Our study coincides with this observation, providing additional evidence that affective distance, an aspect of psychological distance, has a significant correlation with perceived live streamers’ ability. Furthermore, trust is recognized as a vital variable in consumer purchase intention [5,43]. However, the effect of different dimensions of trust on impulsive buying has not been thoroughly explored with adequate research projects [26]. Our research specifies the meaningful impact of perceived live streamers’ ability on trust and contributes further proof for the application of cognitive trust and affective trust in impulsive purchase intention, stressing the crucial mediating effect of affective trust.

### 6.2. Theoretical Implications

Our study investigated the intrinsic formation mechanism of consumer impulsive purchase intention. This investigation offers significant theoretical significance and provides valuable insights for further research.

First, starting from the perceived live streamers’ ability, we validated the impact of perceived live interaction ability on consumer impulsive purchases while emphasizing the crucial significance of affective distance. The research results reveal the influence path of the streamer’s live interaction ability, which enriches the connotation of the research of interaction between streamers and users.

Furthermore, we examined the impact of affective distance between perceived live streamers’ ability and users’ affective trust, broadening the research scenario of affective distance theory. At the same time, the mechanism of perceived linguistic persuasion ability to promote the impulsive purchase intention was explored. Language with apparent persuasive modes significantly impacts consumers’ cognitive and affective reactions. This work introduced Aristotle’s rhetorical appeals into the live-streaming context, achieving the integration of several multidisciplinary theories.

Finally, we explored how two dimensions of trust trigger users’ impulsive purchase intention. The findings reveal that users’ affective trust in live streamers effectively mediates affective distance and impulsive purchase intention, as well as partially mediating cognitive trust and impulsive purchase intention. Expertise, logic, and the morality of perceived linguistic persuasion ability indirectly impact affective trust through cognitive trust and ultimately promote impulsive purchase intention. Our study offers further insight into the mediating mechanism of trust in forming impulsive purchase intention and enriches the scope of live impulsive purchase research.

### 6.3. Practical Implications

Our research provides practical guidance specific to impulsive purchasing behavior. We examined the mechanism of perceived live streamers’ ability to influence consumers’ impulsive purchase intentions, aiming to provide practical suggestions and references for e-commerce consumer research and demonstrate the value of the theoretical application. The specific practical implications are as follows.

According to the study’s results, live streamers can use their interaction ability to influence user perceptions and effectively reduce affective distance, in which responsiveness has the most pronounced effects. This means that the live streamer gives priority to ensuring prompt and immediate connection with viewers, as well as providing timely feedback. Entertainment can be enhanced by incorporating entertainment elements, such as conveying product information to heighten amusement and enjoyment. Additionally, the inclusion of pop-ups, lotteries, voting, and other interactive features can boost audience engagement and enhance the overall live experience. Personalized interaction requires streamers to develop different user programs to meet personalized needs. Specifically, in live interaction, as far as possible, this means understanding the basic information and characteristics of multiple types of users, using own knowledge reserves, etc., to answer users’ questions and create interactive advantages in the live broadcast room.

For perceived linguistic persuasion ability, emotional contagion is a key factor affecting the affective distance, so the recommendation is to increase the proportion of emotion-related words in the live conversation, cut from specific topics, stimulate common emotions, and promote impulsive purchase intentions. Logic has the most significant influence on user cognitive trust, which suggests that streamers can use their logical persuasion ability to elucidate and resolve puzzles for users by utilizing clear logic and precise language, thus increasing their identification. Furthermore, morality has a more significant influence on cognitive trust than expertise, suggesting that users value streamers’ professional knowledge and pay more attention to the streamers’ moral level and personality traits. By establishing trustworthiness and morality, the streamer will promote user trust and effectively convince the user to make a purchase decision.

### 6.4. Limitations and Future Research

Due to the objective research constraints, our research has several limitations. Firstly, we take live streaming as the research purpose, with the respondents mainly involved in e-commerce platforms, including Taobao, Douyin, and Kuaishou. Indeed, the characteristics and user demographics of distinct live-streaming platforms differ. Therefore, we suggest distinguishing the research objects and conducting specific analyses of other platforms to obtain results using personalized research. Secondly, our study adopts a questionnaire survey to convey analysis data. There are particular limits on the quantity and diversity of participants. Hence, the generalizability of the research results should be further considered. The scope might be expanded to collect representative sample data, such as sampling for different countries and populations. In addition, this study collects primary data from users. The analysis focuses on the perceived live streamers’ ability. Subsequent studies can leverage real-time audio and video materials and pop-up comment data generated in live streaming. These data can be analyzed using data mining and machine learning, as well as interview and experimental methods. Furthermore, researchers can incorporate classical theories from other fields to further validate the findings of this study from various perspectives. Finally, this study focused on consumer impulse purchases in relation to live-streaming e-commerce. The validity and suitability of consumer behavior research depend on factors such as the sector, product, or service. In future studies, it is rational to optimize the selection of background, research target, and other factors, thus expanding the application of the results obtained.

## Figures and Tables

**Table 1:** Constructs of perceived linguistic persuasion ability.

Aristotle’s Rhetorical Appeals	Constructs	Definition
Ethos	Morality	Live streamers’ ability to illustrate their moral quality and shape honest images through linguistic expression [27].
	Expertise	Live streamers’ knowledge and ability correlated to commodity trading conveyed by linguistic expression [46].
Logos	Logic	Live streamers’ ability to provide a complete presentation structure and straightforward demonstration process through logical argument [17,28].
Pathos	Emotional contagion	Live streamers’ ability to convey emotions via linguistic expression and cause emotional resonance in consumers [51,52].

**Table 2:** Variable measurement.

Constructs	Items	Measurement	References
Responsiveness (RP)	RP1	The live streamer is pleased to have conversations with me.	Xue et al. [32]
	RP2	The live streamer can swiftly reply to my questions.	
	RP3	The live streamer’s responses are tightly associated with my questions.	
	RP4	The live streamer can instantly provide pertinent information according to my questions.	
Entertainment (ET)	ET1	The live streamer launches an exciting spike activity to attract me to interact with him.	Xue et al. [32]
	ET2	The live streamer explains the product details interestingly, including funny ways of utilizing it.	
	ET3	In the live streaming room, I enjoy shopping by entertaining social activities related to the products or services with streamers.	
Personalization (PI)	PI1	The live streamer will provide me with professional advice based on my product browsing.	Xue et al. [32]
	PI2	The live streamer will pay attention to my requirements for products and services.	
	PI3	The live streamer will satisfy me with unique product recommendations that align with my requirements.	
Emotional contagion (EC)	EC1	In live streaming, what the live streamer says can move my emotions.	Reniers et al. and Shen [51,52]
	EC2	In live streaming, streamers’ words can touch me.	
	EC3	In live streaming, streamers’ words can make me feel the same emotions she/he does.	
Expertise (EP)	EP1	The live streamer recommends goods or services with detailed explanations, making it seem like he/she is knowledgeable.	Johnson and Grayson [46]
	EP2	The live streamer recommends goods or services in fluent language and makes it seem like he/she understands the product.	
	EP3	The live streamer uses specialized vocabulary during the live broadcast, giving the impression that he/she is a connoisseur.	
Logic (LO)	LO1	Generally speaking, the live streamer speaks with reason and evidence.	Chang [28]
	LO2	Generally speaking, the live streamer speaks clearly.	
	LO3	Generally speaking, the live streamer’s speech is logical.	
	LO4	Generally speaking, what the live streamer says is reasonable.	
Morality (MO)	MO1	The way the live streamer talks makes people think he/she is an upright person.	Zhou [27]
	MO2	The way the live streamer talks makes people think he/she is a frank person.	
	MO3	The way the live streamer talks makes people feel that he/she is a sincere person.	
	MO4	The way the live streamer talks makes people think he/she is a friendly person.	
Affective distance (AD)	AD1	In live streaming, I sense that live streamers have an affinity.	Lu et al. [24]
	AD2	In live streaming, I sense that live streamers are likeable.	
	AD3	In live streaming, I sense that live streamers can resonate with my emotions.	
Cognitive trust (CT)	CT1	I think streamers are professional and dedicated during live broadcasts.	McAllister [45]
	CT2	Given the past performance of the live streamer, I have no reason to doubt their ability to live stream and promote sales.	
	CT3	In live streaming, I will rely on recommendations from the live streamer for shopping.	
	CT4	I am full of confidence in the professional ability of the live streamer.	
	CT5	I think most people, even if they are not fans of live streamers, trust live streamers’ professional abilities.	
Affective trust (AT)	AT1	The live streamer shows a warm and caring attitude towards the audience.	Srivastava et al. [30]
	AT2	When the audience has questions about the product, I’m convinced the live streamer will reply seriously to them.	
	AT3	When the audience has questions about the product, I’m convinced the live streamer will actively respond to them.	
	AT4	I can freely ask the live streamer questions through bullet comments and know that the live streamer is willing to view these questions.	
Impulsive purchase intention (IPT)	IPT1	When I watch live e-commerce broadcasts, I have an impulse to buy items that weren’t listed in the original purchasing plan.	Parboteeah et al. [9]
	IPT2	When I watch live e-commerce broadcasts, I possess an intense desire to buy items that weren’t listed in the original purchasing plan.	
	IPT3	When I watch live e-commerce broadcasts, I possess a tendency to buy items that weren’t listed in the original purchasing plan.	

**Table 3:** Sample demographics.

Variable	Classification	Population	Percentage
Gender	Male	147	44.55%
	Female	183	55.45%
Education	High school degree and below	4	1.21%
	Bachelor’s degree	301	91.21%
	Master’s degree and above	25	7.58%
Age	18–25	95	28.79%
	26–35	182	55.15%
	36–45	42	12.73%
	>46	11	3.33%

**Table 4:** Cronbach’s alpha and convergent validity.

Constructs	Items	Loading	T-Values	CR	AVE	Cronbach’s Alpha
Responsiveness (RP)	RP1	0.774	29.743	0.864	0.613	0.79
	RP2	0.791	32.827			
	RP3	0.803	34.583			
	RP4	0.763	26.641			
Entertainment (ET)	ET1	0.835	31.382	0.885	0.72	0.807
	ET2	0.837	34.418			
	ET3	0.874	43.249			
Personalization (PI)	PI1	0.85	41.547	0.847	0.649	0.731
	PI2	0.783	17.792			
	PI3	0.782	24.19			
Emotional contagion (EC)	EC1	0.856	53.539	0.858	0.668	0.754
	EC2	0.751	22.939			
	EC3	0.841	51.693			
Expertise (EP)	EP1	0.892	48.89	0.908	0.766	0.849
	EP2	0.841	32.597			
	EP3	0.891	56.41			
Logic (LO)	LO1	0.788	32.248	0.858	0.603	0.78
	LO2	0.781	26.372			
	LO3	0.77	29.481			
	LO4	0.766	27.554			
Morality (MO)	MO1	0.78	30.061	0.853	0.593	0.771
	MO2	0.764	26.937			
	MO3	0.739	22.212			
	MO4	0.795	31.647			
Affective distance (AD)	AD1	0.857	46.799	0.899	0.747	0.831
	AD2	0.853	37.358			
	AD3	0.883	66.379			
Cognitive trust (CT)	CT1	0.716	27.055	0.858	0.55	0.793
	CT2	0.829	43.003			
	CT3	0.702	18.048			
	CT4	0.811	47.115			
	CT5	0.634	14.582			
Affective trust (AT)	AT1	0.872	52.957	0.908	0.712	0.865
	AT2	0.835	34.585			
	AT3	0.829	39.03			
	AT4	0.839	40.54			
Impulsive purchase intention (IPT)	IPT1	0.867	45.118	0.902	0.754	0.837
	IPT2	0.876	55.597			
	IPT3	0.861	47.428			

**Table 5:** Fornell–Larcker criterion.

Constructs	RP	ET	PI	EC	EP	LO	MO	AD	CT	AT	IPT
RP	0.783										
ET	0.426	0.849									
PI	0.547	0.367	0.806								
EC	0.476	0.415	0.399	0.818							
EP	0.397	0.394	0.431	0.448	0.875						
LO	0.471	0.429	0.492	0.548	0.458	0.776					
MO	0.46	0.418	0.443	0.553	0.388	0.585	0.770				
AD	0.561	0.441	0.469	0.552	0.482	0.631	0.6	0.865			
CT	0.501	0.369	0.451	0.592	0.432	0.635	0.544	0.65	0.742		
AT	0.497	0.368	0.438	0.485	0.401	0.376	0.386	0.487	0.466	0.844	
IPT	0.244	0.297	0.207	0.464	0.430	0.276	0.341	0.359	0.412	0.564	0.868

**Table 6:** Cross-loadings.

Constructs	RP	ET	PI	EC	EP	LO	MO	AD	CT	AT	IPT
RP1	0.774	0.323	0.446	0.39	0.318	0.409	0.327	0.41	0.373	0.363	0.166
RP2	0.791	0.357	0.342	0.328	0.315	0.269	0.273	0.409	0.321	0.382	0.206
RP3	0.803	0.345	0.456	0.336	0.288	0.396	0.417	0.462	0.437	0.38	0.145
RP4	0.763	0.31	0.459	0.434	0.323	0.394	0.408	0.47	0.427	0.426	0.244
ET1	0.369	0.835	0.302	0.35	0.34	0.391	0.328	0.341	0.287	0.333	0.254
ET2	0.349	0.837	0.335	0.379	0.391	0.358	0.409	0.365	0.352	0.276	0.291
ET3	0.367	0.874	0.299	0.332	0.28	0.349	0.329	0.413	0.303	0.328	0.218
PI1	0.47	0.333	0.85	0.316	0.354	0.442	0.384	0.43	0.403	0.407	0.139
PI2	0.391	0.276	0.783	0.308	0.323	0.386	0.365	0.362	0.367	0.32	0.196
PI3	0.462	0.271	0.782	0.346	0.37	0.352	0.317	0.334	0.312	0.323	0.173
EC1	0.433	0.388	0.336	0.856	0.429	0.485	0.499	0.491	0.538	0.463	0.441
EC2	0.251	0.292	0.26	0.751	0.325	0.325	0.364	0.346	0.322	0.283	0.281
EC3	0.451	0.332	0.368	0.841	0.339	0.507	0.476	0.495	0.554	0.418	0.395
EP1	0.377	0.358	0.4	0.397	0.892	0.436	0.332	0.454	0.415	0.355	0.363
EP2	0.304	0.324	0.369	0.382	0.841	0.396	0.365	0.366	0.306	0.346	0.373
EP3	0.353	0.349	0.363	0.398	0.891	0.373	0.332	0.436	0.398	0.354	0.397
LO1	0.419	0.43	0.418	0.48	0.404	0.788	0.452	0.527	0.488	0.354	0.251
LO2	0.321	0.365	0.346	0.402	0.322	0.781	0.466	0.473	0.492	0.322	0.243
LO3	0.319	0.292	0.365	0.392	0.332	0.77	0.477	0.438	0.467	0.226	0.167
LO4	0.4	0.249	0.396	0.426	0.363	0.766	0.424	0.517	0.521	0.264	0.194
MO1	0.376	0.296	0.368	0.404	0.283	0.437	0.78	0.506	0.433	0.338	0.264
MO2	0.339	0.354	0.326	0.488	0.349	0.488	0.764	0.48	0.451	0.309	0.273
MO3	0.296	0.285	0.309	0.399	0.193	0.445	0.739	0.396	0.363	0.219	0.198
MO4	0.399	0.345	0.359	0.407	0.355	0.43	0.795	0.455	0.42	0.312	0.309
AD1	0.514	0.396	0.416	0.505	0.44	0.577	0.579	0.857	0.557	0.458	0.314
AD2	0.448	0.374	0.432	0.441	0.359	0.507	0.423	0.853	0.566	0.351	0.295
AD3	0.489	0.373	0.37	0.482	0.446	0.548	0.543	0.883	0.565	0.447	0.322
CT1	0.443	0.243	0.41	0.452	0.297	0.505	0.41	0.463	0.716	0.392	0.307
CT2	0.359	0.355	0.395	0.55	0.41	0.539	0.465	0.559	0.829	0.365	0.387
CT3	0.295	0.336	0.262	0.429	0.349	0.34	0.299	0.432	0.702	0.374	0.33
CT4	0.369	0.271	0.337	0.422	0.279	0.528	0.437	0.524	0.811	0.327	0.309
CT5	0.39	0.152	0.238	0.318	0.262	0.414	0.391	0.42	0.634	0.265	0.176
AT1	0.408	0.384	0.386	0.413	0.379	0.299	0.325	0.415	0.413	0.872	0.499
AT2	0.457	0.316	0.375	0.428	0.384	0.306	0.319	0.415	0.374	0.835	0.444
AT3	0.391	0.241	0.394	0.352	0.334	0.299	0.314	0.387	0.361	0.829	0.528
AT4	0.422	0.3	0.32	0.448	0.253	0.366	0.347	0.428	0.425	0.839	0.426
IPT1	0.234	0.337	0.209	0.377	0.457	0.241	0.293	0.329	0.343	0.473	0.867
IPT2	0.2	0.177	0.138	0.378	0.304	0.222	0.258	0.331	0.35	0.517	0.876
IPT3	0.202	0.267	0.196	0.457	0.365	0.257	0.342	0.274	0.383	0.477	0.861

**Table 7:** Result of mediation test.

Independent Variable (IV)	Mediating Variable (M)	Dependent Variable (DV)	IV→DV (a)	IV→M (b)	IV + M→DV		Conclusion
					M→DV	IV→DV	
					(c)	(d)	
Affective distance	Affective trust	Impulsive purchase intention	0.361 ***	0.490 ***	0.510 ***	0.111 ^NS^	Complete mediation
Cognitive trust	Affective trust	Impulsive purchase intention	0.423 ***	0.474 ***	0.471 ***	0.198 ***	Partial mediation

Notes: *** *p* < 0.001; ^NS^: non-significant at the 0.05 level.

## Data Availability

The data presented in this study are available on request from the corresponding authors.
